# Estimating the burden of vaccine-preventable lower respiratory tract disease in UK primary care: protocol for a prospective surveillance study (AvonCAP GP2)

**DOI:** 10.3399/BJGPO.2024.0129

**Published:** 2024-12-18

**Authors:** Polly Duncan, Ruth Mears, Elizabeth Begier, Sanaz Rouhbakhsh Halvaei, Jo Southern, Siân Bodfel Porter, Robin Hubler, Glenda Oben, George Qian, Maria Lahuerta, Tim Davis, James Campling, Shoba Dawson, Hannah Christensen, Jennifer Oliver, Begonia Morales-Aza, Kaijie Pan, Sharon Gray, Catherine Hyams, Leon Danon, Bradford D Gessner, Adam Finn, Alastair D Hay, AvonCAP GP2 research group

**Affiliations:** 1 Centre for Academic Primary Care, University of Bristol, Bristol, UK; 2 Bristol Vaccine Centre, Schools of Population Health Science and Cellular and Molecular Medicine, University of Bristol, Bristol, UK; 3 Global Respiratory Vaccines, Medical & Scientific Affairs, Pfizer Inc, Collegeville, PA, US; 4 Evidence Generation, Pfizer Inc, Collegeville, PA, US; 5 Vaccines Medical Affairs, Pfizer Ltd, Tadworth, UK; 6 EvGen Statistics, Pfizer Research and Development, Pfizer Inc, Collegeville, PA, US; 7 School of Engineering Mathematics and Technology, University of Bristol, Bristol, UK; 8 West of England NIHR Clinical Research Network, Bristol, UK

**Keywords:** primary health care, lower respiratory tract infection, respiratory tract diseases, respiratory syncytial viruses

## Abstract

**Background:**

The true burden of acute lower respiratory tract disease (aLRTD; includes acute lower respiratory tract infection [aLRTI] and presumed non-infective exacerbations of chronic lung disease and heart failure) among adults presenting to primary care, and the proportion that are potentially vaccine preventable is unknown.

**Aim:**

To describe aLRTD incidence in adults presenting to primary care; estimate proportions caused by respiratory syncytial virus (RSV), severe acute respiratory syndrome coronavirus 2 (SARS-CoV-2), and Streptococcus pneumoniae (SP); and investigate disease burden from patient and NHS perspectives.

**Design & setting:**

Primary care prospective cohort study conducted in six representative general practices (total ∼86 000 registered adults) in Bristol, UK.

**Method:**

Adults (aged ≥18 years) registered at participating general practices and presenting to primary care (in-hours or out-of-hours) or emergency department (if not admitted) with aLRTD will be eligible. They will be identified by real-time primary care record searches. Researchers will screen electronic GP records, including free text, contact patients to assess eligibility, and offer enrolment in a surveillance study and an enhanced diagnostic study (urine, saliva, and respiratory samples; physical examination; and symptom diaries). Data will be collected for all aLRTD episodes, with patients assigned to one of three arms: surveillance; embedded diagnostic; and descriptive dataset. Outcome measures will include clinical and pathogen-defined aLRTD incidence rates, symptom severity and duration, NHS contacts and costs, health-related quality-of-life changes, and mortality (≤30 days post-identification).

**Conclusion:**

This comprehensive surveillance study of adults presenting to primary care with aLRTD, with embedded detailed data and sample collection, will provide an accurate assessment of aLRTD burden due to vaccine-preventable infections.

## How this fits in

There is a paucity of evidence, both in the UK and globally, regarding the true burden and aetiology of acute lower respiratory tract infection (aLRTI) among adults presenting to primary care. In this study, we will prospectively screen adults registered with one of six general practices in Bristol, UK, and identify those with acute lower respiratory tract disease (aLRTD; including aLRTI and presumed non-infective exacerbations of chronic lung disease and heart failure). For eligible patients, data will be extracted from electronic GP records and, for a subset, samples and additional data will be collected, including examination findings, surveys, and symptom diaries. Study findings will estimate the incidence and burden of aLRTI for adults presenting to primary care, and the proportion that could potentially be prevented by vaccines. Data will be combined with a sister study of hospitalised adults to estimate the overall burden of aLRTI across primary and secondary care.

## Introduction

Vaccines against respiratory pathogens *Streptococcus pneumoniae* (SP), respiratory syncytial virus (RSV), and severe acute respiratory syndrome coronavirus 2 (SARS-CoV-2) are licensed in the UK. Traditionally, the burden of vaccine-preventable illness has been estimated by studying the incidence of severe illness in secondary care.^1,2^ While important in terms of morbidity and mortality, this underestimates the total disease and societal burden. This impacts calculations of vaccine cost-effectiveness since a high community incidence can offset the lower costs associated with less severe illness.

The healthcare cost of hospitalised community-acquired pneumonia (CAP) across Europe and in the UK before the SARS-CoV-2 pandemic was estimated at £8.6 billion and £731 million per annum, respectively.^3,4^ The incidence of aLRTIs and CAP in Europe varies by country, age, and sex; however, in all studies incidence increased markedly with age.^5–9^ A large UK study of older adults (aged ≥65 years) presenting to primary and secondary care reported annual incidences of CAP and aLRTI of 8/1000 and 123/1000 person–years, respectively, although this study relied on retrospective review of coded data.^9^

The true burden and aetiology of aLRTIs within UK primary care is unknown,^1,10^ but it is important to ascertain the potential benefit of vaccines to reduce disease burden for the patient (for example, time off work and quality-of-life loss),^11^ the NHS (for example, healthcare utilisation costs and system capacity), and society (for example, lost productivity costs). Furthermore, vaccinating ‘at-risk’ cohorts may help reduce antimicrobial resistance.^12^ Partial burden estimates are available in the UK through: (i) routinely collected primary care data (but limited by incomplete and non-standardised coding, and lack of microbiological data);^6,9^ and (ii) bespoke prospective cohort studies (limited by recruitment selection bias and failure to collect data on patients not sampled).^13,14^ A recent study investigating the appropriateness of antibiotic prescriptions in UK primary care found that 37% of antibiotics prescribed were not linked to a diagnostic code.^15^

Typically, people with aLRTI present to primary care with acute cough and symptoms or signs attributable to lower respiratory tract involvement, including sputum production, wheeze, or shortness of breath. However, evidence suggests that exacerbations of heart failure (HF) and chronic lung diseases may be triggered by similar microbes in the absence of typical ‘infection’ symptoms. Here, we use the term ‘acute lower respiratory tract disease’ to describe both aLRTI (for example, pneumonia, acute bronchitis, and infective exacerbation of chronic lung disease) and exacerbations of HF and chronic lung disease presumed to be ‘non-infective’.

### Aim

This study aims to describe the incidence and burden of aLRTD in adults (aged ≥18 years) presenting to primary care in Bristol, and estimate the proportion caused by vaccine-preventable infections, including SP, RSV, and SARS-CoV-2.

### Objectives

- Describe the demographic, clinical, and microbiological characteristics for adults presenting to primary care with aLRTD.
- Investigate the natural history of aLRTD, including patient-reported symptom duration and severity; antibiotic and antiviral consumption; respiratory pathogen isolation; time off work, primary care consultations, hospital admission, and quality of life for up to 12 months.
- Describe time trends in population-based incidence rates of aLRTD.
- Determine mortality rate at 30 days (and up to 12 months for those who have not recovered at 28 days) after primary care visit for aLRTD.
- Determine the pathogen distribution rates of RSV, SARS-CoV-2, and other viral pathogens among adults diagnosed with exacerbation of heart failure and non-infective exacerbation of chronic lung disease, including asthma and chronic obstructive pulmonary disease (COPD).
- Estimate the financial costs of aLRTD from NHS perspectives.
- Describe the proportion of presumed non-infective aLRTD diagnoses associated with respiratory pathogens.

## Method

### Study design and setting

**Figure 1.**
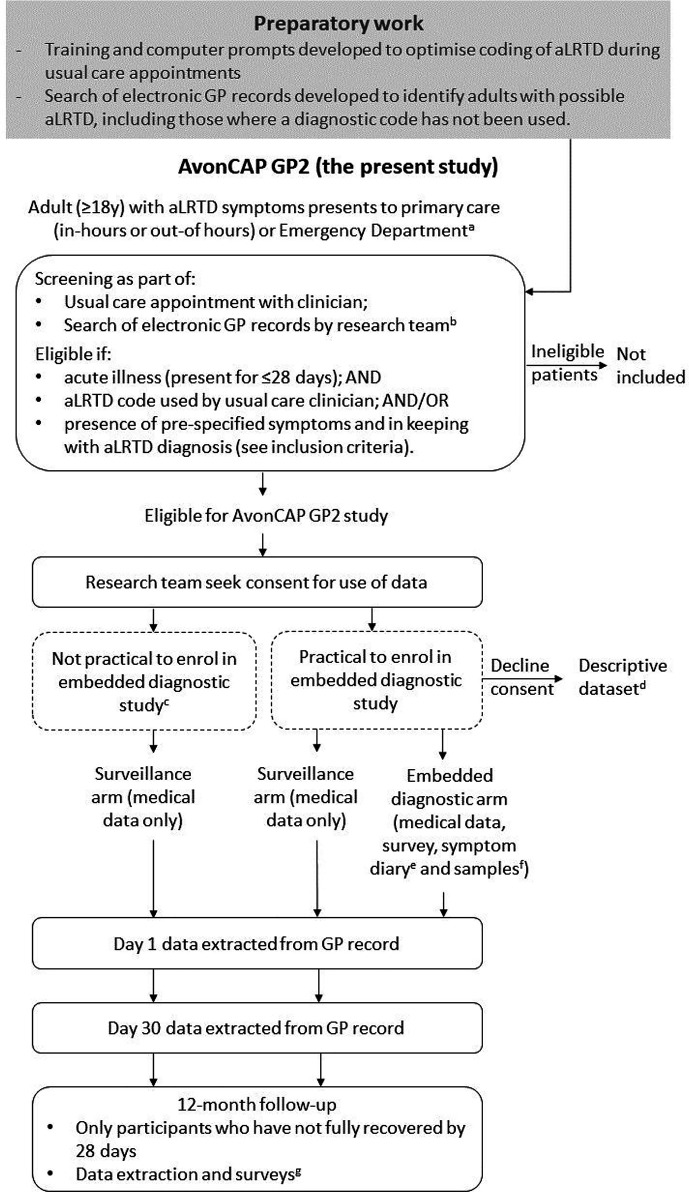
Study flow diagram ^a^For adults who present to the emergency department, they will only be eligible if they are not admitted to hospital. ^b^The research team will run a search of the electronic GP records at least twice a day to identify adults with aLRTD diagnosis, symptoms, clinical findings, and/or prescribed antibiotics. They will manually review the consultation notes, including free text, and contact the patient to assess eligibility. ^c^We have approval from the Confidentiality Advisory Group to include specific groups of patients in the surveillance arm without consent, including those we are unable to contact (at least three failed contact attempts over ≥24 hours) and patients at the end of life. If a patient lacks capacity to provide consent (for example, owing to cognitive impairment or dementia), the research team will attempt to contact a family member, friend, or unpaid carer of the patient (that is, a personal consultee) to discuss the patient’s willingness to take part. If they are unable to contact a personal consultee or if the person contacted does not want to take on the role of personal consultee, the research practitioner or nurse will attempt to identify and contact a nominated consultee. ‘Medical data’ refers to routinely collected data within the electronic GP records. ^d^If a patient declines consent (or other specified cohorts of patient), descriptive data will be collected, including: screening ID, anonymous encrypted NHS number, date of eligible condition, reasons for declining to participate, eligibility, qualifying condition, severity of illness, gender, age, deprivation decile, ethnicity, whether they reside in a care home or not, whether they require a translator (and if so what language), category of patient presentation (out-of-hours GP, in-hours GP, or emergency department), whether they die within 30 days of their appointment, and, if so, whether their death was related to their qualifying illness. ^e^Daily symptom diary until recovery from illness or for up to 28 days. ^f^Nose and/or throat, saliva, and urine samples collected on day 1. ^g^Follow-up survey (on quality of life, time off work, and recovery) at 6 weeks, 8 weeks, 3 months, 4 months, 5 months, 6 months, 9 months, and 12 months (until fully recovered from illness). aLRTD = acute lower respiratory tract disease.

This prospective cohort study will take place in Bristol, UK, from 14 February 2022–31 July 2024. Six general practices (total ∼86 000 registered adult patients), serving populations with varying sociodemographics (deprived or affluent, urban or semi-urban, and different ethnic groups) and covering a wide geographical area, will be participating (see Supplementary Table S1). Figure 1 shows the study summary.

### Recruitment

To enable comprehensive surveillance, potential participants will be identified, screened, and invited to take part prospectively each day (Monday to Friday).

### Eligibility criteria

Adults (aged ≥18 years) registered at one of the six practices will be eligible if they present to the practice, out-of-hours (OOH) primary care (one provider in Bristol), or the emergency department (ED, if not admitted to hospital) with an acute illness (onset or worsening of symptoms ≤28 days) and symptoms or signs consistent with a clinical aLRTD diagnosis. The full inclusion and exclusion criteria are shown in Box 1.

Box 1 **Eligibility criteria**

**Inclusion criteria (all must be met)**

1. Aged ≥18 years

2. Presenting to primary care with acute illness (symptoms present for ≤28 days)

3. Evidence of aLRTD^a^ as guided by the following criteria:

 1. Evidence of aLRTI (including acute bronchitis, pneumonia, and infective exacerbations of chronic lung disease):

  i. clinical suspicion of LRTI and new/worsened cough with ≥1 of following signs/symptoms: sputum production or purulence, chest pain,

  wheeze, shortness of breath, tachypnoea, or abnormal auscultatory findings suggestive of aLRTI; OR

  ii. clinical diagnosis of aLRTI.

 OR

 2. Evidence of acute exacerbation of pre-existing heart failure with respiratory symptoms:

  i. clinical suspicion of acute exacerbation of pre-existing heart failure and new or worsening of ≥2 of following signs/symptoms: cough

  (including nocturnal cough), shortness of breath, wheeze, tachypnoea, or abnormal auscultatory findings suggestive of exacerbation of heart   failure; OR

  ii. clinical diagnosis of acute exacerbation of heart failure with respiratory symptoms.

 OR

 3. Evidence of non-infective exacerbation of pre-existing chronic lung disease:

  i. clinical suspicion of presumed non-infective exacerbation of pre-existing chronic lung disease and new or worsening of ≥2 of following

  signs/symptoms: cough, shortness of breath, wheeze, tachypnea, or abnormal auscultatory findings suggestive of acute non-infective   exacerbation; OR

  ii. clinical diagnosis of non-infective exacerbation.

**Exclusion criteria (if any met)**

1. Previously enrolled participants within 28 days of the onset of the study qualifying aLRTD illness.

2. At the time of enrolment, alternative non-LRTD working diagnosis suspected.

3. Presenting to primary care with the same episode of aLRTD for which they have been discharged from hospital.

4. Any patient who develops signs and symptoms of LRTD after being hospitalised for ≥48 hours and is within 7 days of discharge from hospital.

^a^Acute lower respiratory tract disease includes acute lower respiratory infection (including acute bronchitis, pneumonia and infective exacerbations of chronic lung disease), acute exacerbation of heart failure and presumed non-infective exacerbation of pre-existing lung disease. aLRTD = acute lower respiratory tract disease. aLRTI = acute lower respiratory tract infection. LRTD = lower respiratory tract disease. LRTI = lower respiratory tract infection.

### Optimising the use of diagnostic codes

In UK primary care, clinicians document consultations by using symptom or diagnostic codes and/or by entering ‘free-text’ notes into the electronic GP records. Respiratory infections are generally poorly coded, with clinicians reluctant to use diagnostic codes (for example, ‘acute lower respiratory tract infection’) and preferring to use symptom codes (for example, ‘cough’) or to not provide a code, even when they suspect an infection.^16^ To mitigate this, we have developed and implemented interventions (training and computer prompts) to optimise coding of aLRTD.

For adults with symptoms or clinical signs of respiratory infections and/or prescribed antibiotics, an electronic prompt will appear, asking whether the patient has a suspected aLRTD with onset of symptoms ≤28 days (see Supplementary Appendix S1). For those who select ‘yes’, a template will appear asking the clinician to select the relevant diagnostic code from a drop-down list and to rate the severity of the illness (mild, moderate, or severe). The clinicians will be able to send a pre-prepared text message and/or briefly discuss the study with patients, recording their action in the template. Patients who do not want to be contacted by the research team will be recorded by the clinician as having declined consent.

### Identifying potential participants

Recognising that use of diagnostic aLRTD codes will be suboptimal, a search of the electronic GP records system has been developed to identify all adults with possible aLRTD, including those where a diagnostic code has not been used. The search identifies adults: 1) with a clinical diagnosis of aLRTD; 2) with pre-existing asthma or COPD prescribed an antibiotic or oral steroid; 3) with pre-existing HF prescribed a diuretic; or 4) where respiratory symptoms (cough, shortness of breath, wheeze, and/or chest pain) have been coded or an antibiotic used to treat aLRTI has been prescribed. The search will be run by the research team at least twice a day during the normal working week (Monday to Friday). Eligible patients presenting to OOH primary care or ED will be identified through letters documented in their primary care records.

### Screening

A researcher will manually review the electronic GP records of all potentially eligible patients, including free text, to assess eligibility against defined criteria (Box 1), and contact the patient to further assess eligibility and invite them to take part.

### Inclusivity and consent

Online, telephone, postal, or face-to-face consent will be taken, depending on patient preference. Patients will be invited to consent to the surveillance arm only (i.e. electronic GP record data) or the embedded diagnostic arm (i.e. electronic GP record data plus samples, surveys, or examination findings). To improve inclusivity, patients will be offered standard or ‘easy read’ versions of the information sheets and consent forms. Patients will be encouraged to watch two short films: a generic ‘Why take part in research?’ and a further explaining study participation specifics. For non-English-speaking patients, study documents and short films have been translated into 10 commonly spoken languages in Bristol. For patients who lack capacity to consent, we will endeavour to identify a consultee to determine the patient’s willingness to take part and, where appropriate, obtain a consultee declaration.

### Data collection

Data will be collected for all aLRTD episodes, with patients assigned to one of three study arms:

- a surveillance arm;
- an embedded diagnostic arm; and
- a descriptive dataset arm.

#### Surveillance arm

The index consultation date will be defined as the date the participant appears in the electronic GP record search (in-hours patients) and is assessed as eligible; or the attendance date (OOH and ED patients).

Information about sociodemographics, qualifying condition, severity of illness (mild, moderate, or severe), smoking status, pregnancy status, vaccination history (SARS-CoV-2, influenza, and/or pneumococcus) and long-term conditions will be collected (Table 1). Health utility data (hospital admission, ED attendances, outpatient appointments, in-hours and OOH primary care appointments, 111 calls [NHS phone triage system], and ambulance attendances) and final diagnosis will be collected for the 30-day period after the index consultation date.

**Table 1.** Summary of variables to be collected

Descriptive dataset arm	
Sociodemographics	Age Gender Deprivation decile Ethnicity (White and Black, Asian, and Minority ethnic) Care home resident Translator required (if so, what language)
Illness episode	Clinical diagnosis Severity of acute illness (reported by clinician) Date of eligible condition Category of patient presentation (in-hours or out-of-hours primary care, or emergency department) Date of death and whether the death was related to the qualifying illness
**Surveillance arm (data collected in addition to descriptive dataset arm variables)**	
General health	Long-term conditions^a^ Smoking status Alcohol status Pregnancy status Rockwood Frailty Scale score Risk factors for pneumococcal infection
Vaccinations	Influenza SARS-CoV-2 Pneumococcal
Symptoms	Upper and lower respiratory symptoms Heart failure symptoms
Complications	Acute renal failure, liver dysfunction, myocardial infarction, new atrial fibrillation, stroke, transient ischaemic attack, deep vein thrombosis, pulmonary embolus, new or worsening of congestive heart failure, fall, reduced mobility, and/or increased care requirements
Primary care attendances	Number and type of primary care appointments (in-hour and out-of-hours)
Secondary care attendances	Hospital admission (length of stay, treatment, and outcome) Outpatient appointments
Use of other healthcare services	Emergency department attendances 111 calls Ambulance attendances
Investigations	Radiology (for example, CXR and CT scan) Microbiology SARS-CoV-2 test results
Treatment	Antibiotics Steroids Inhalers Diuretics Antivirals
Vital status	Date of death (if occurring within 30 days of index consultation date or for up to a maximum of 12 months for those who have not recovered at 28 days)
**Embedded diagnostic arm (data collected in addition to descriptive dataset and surveillance variables**)	
Surveys	EQ-5D-5L (today, worst day of illness, and before this illness) Number of children and adults living in the household Employed as a healthcare worker
Samples	Nose and/or throat swab Saliva Urine
Physical examination	Confusion screen^b^ Temperature^b^ Oxygen saturation^b^ Heart rate^b^ Respiratory rate^b^ Blood pressure^b^ Weight Height Body mass index
Symptom diary (until fully recovered from illness for up to 28 days)	Presence and severity of symptoms^17^ (completed daily) EQ-5D-5L (completed weekly) Time off work (completed weekly)

^a^Long-term conditions from the 20 medical conditions included in the Cambridge Multimorbidity Score^19^ and the Charlson Comorbidity Index score.^20^ ^b^Examination findings collected from February 2022–October 2023 only. CT = computed tomography. CXR = chest radiography.

#### Embedded diagnostic arm

In addition to collecting the surveillance arm data, a research visit will be conducted to collect: samples (nose and/or throat swab, saliva, and urine); examination findings; surveys; and/or day one of a 28-day symptom diary (Table 1). Health-related quality-of-life measures will also be assessed (EQ-5D-5L).

For the symptom diary, participants can choose to complete an online version, paper version, or to receive daily telephone calls from the research team. A validated measure will record presence and severity of symptoms,^17^ recorded daily using a scale from 0 (no problem) to 6 (as bad as it could be). Weekly questions about quality of life (EQ-5D-5L),^18^ recovery of illness, and time off work will be included. The symptom diary will be completed until resolution of symptoms for up to 28 days. Participants who have not fully recovered by 28 days will be invited to complete follow-up diaries (EQ-5D-5L and time off work) at 6 weeks and 8 weeks, then 3 months, 4 months, 5 months, 6 months, 9 months, and 12 months until they have fully recovered.

Participants will be reimbursed with vouchers for each research visit (£20) and symptom diary (£20).

#### Descriptive dataset

With ethical approval, a small dataset will be collected for participants who decline consent and other specified groups (Figure 1). Data collected: age, gender, deprivation decile, ethnic group, whether the patient resides in a care home, whether they require a translator (and if so what language), clinical diagnosis, severity of illness, date of eligible condition and category of presentation (in-hours or OOH primary care, or ED). For patients who die within 30 days of the index consultation, we will collect the date of death and a clinician will ascertain whether the death was related to their qualifying illness.

### Analysis plan

Analyses will be based on available data pooled across participating general practices and, unless otherwise indicated, will be characterised based on the collated demographic, clinical, and epidemiological variables (described above). The cohort will describe aLRTD, stratified by clinical diagnosis, as appropriate.

Continuous data will be described using median and interquartile range or mean and standard deviation for all variables collected from the entire cohort or specific subgroups. Categorical variables will be described using frequencies and proportions. The incidence of aLRTD will be calculated as the number of eligible cases divided by total number of adults registered at participating general practices and presented as per 1000 person–years. Pathogen-specific rates will be calculated as the number testing positive divided by the total number tested and presented as percentages.

Specific analyses, including survival analysis, and univariate and multivariable regression models will be used to explore the relationships between aLRTD and known risk factors (covariates), as appropriate. In addition, details of analytical methodology applied will be provided for each analysis. Handling of missing values will be decided based on the type and frequency of these. Results will be presented with 95% confidence intervals and, where applicable, *P*-values will be used to indicate statistical significance.

## Discussion

### Summary

In this comprehensive surveillance study, we will prospectively identify and collect data on adults presenting to primary care with aLRTD. For a subset of adults, we will collect nose and/or throat, saliva and urine samples, examination findings, surveys, and health utility data.

### Strengths and limitations

A key strength of the study is the rigorous approach to identifying eligible patients and collection of data for all episodes of aLRTD, ensuring accurate incidence estimates. Potentially eligible patients will be identified using a search of electronic GP records, including patients where a diagnostic code has not been used. The electronic GP records will be screened, including free-text consultation notes, and patients contacted prospectively where feasible to establish eligibility. These strategies will overcome a limitation of existing retrospective studies, which rely on routinely collected coded primary care data that can lead to inaccurate incidence estimates, especially for infections.^15^ Furthermore, all patients we contact will be invited to provide samples and surveys as part of the embedded diagnostic study. Lastly, in this study, incidence denominators will be derived from the list of patients registered at participating general practices. In the UK, as residents cannot register with multiple practices, the denominator can be accurately determined. This overcomes a limitation of other studies, resulting in more accurate incidence estimates.

One limitation is that our searches rely on symptoms and diagnoses being coded or treatment prescribed, so some instances of aLRTD where the clinician has recorded only free text and not prescribed treatment will be missed. However, we have mitigated this through interventions and training as part of a previous study. A further limitation is the generalisability of the results, in that data will be collected from a small number of general practices in one geographical area. However, we have selected practices serving populations with varying sociodemographic characteristics, and our analytic approach is designed to provide findings that are generalisable to the wider UK population.

### Implications for practice

This study will provide accurate estimations of the incidence and burden of aLRTD for adults presenting to primary care in the UK. These data, combined with findings from a sister study of hospitalised adults, will provide important evidence to policymakers about the potential benefit of vaccines to reduce the burden of aLRTDs.
